# Biopsy Diagnosis Challenges in Oral Carcinoma Cuniculatum: A Case Report

**DOI:** 10.7759/cureus.100868

**Published:** 2026-01-05

**Authors:** Shingo Kodama, Takayuki Kurimoto, Takasuke Usuki, Yuichiro Hamamoto, Shoichiro Ishii

**Affiliations:** 1 Department of Oral and Maxillofacial Surgery, Osaka University, Suita, JPN; 2 Department of Oral and Maxillofacial Surgery, Kinki Central Hospital, Itami, JPN; 3 Department of Pathology, Kinki Central Hospital, Itami, JPN

**Keywords:** biopsy, fistula, gingiva, keratin discharge, oral carcinoma cuniculatum

## Abstract

Oral carcinoma cuniculatum (OCC) is an extremely rare variant of squamous cell carcinoma that often poses diagnostic difficulties on biopsy despite clinical indications of malignancy. We report the case of a 90-year-old woman who presented with a granular endophytic lesion on the maxillary gingiva, accompanied by numerous fistulas exuding white, keratin-like material. Computed tomography revealed marked bone resorption. Although two initial biopsies were inconclusive, a third biopsy targeting deeper tumor tissue adjacent to the resorbed bone confirmed the diagnosis of OCC. The presence of keratin-like discharge from the tumor may be a diagnostic clue, and obtaining tissue from deeply infiltrated regions is essential for accurate diagnosis.

## Introduction

Oral carcinoma cuniculatum (OCC) is a low-grade variant of squamous cell carcinoma (SCC) characterized by histopathological features resembling rabbit burrows, as reported by Flieger et al. in 1977 [1]. OCC is an extremely rare entity; according to a recent systematic review, only 115 cases have been reported in the literature to date [2]. Clinically, OCC typically presents as a slow-growing, broad-based mass with a pink, red, or white cobblestone-like mucosal surface and a nodular appearance, and it can locally invade the surrounding soft tissues. Moreover, OCC has a strong propensity for bone invasion [2,3]. Although the disease exhibits clinical findings strongly suggestive of malignancy, establishing a definitive diagnosis based on histopathological evaluation of routine biopsy specimens, which are typically obtained from the superficial portion of the tumor, remains challenging; accordingly, only 29.1% of cases were diagnosed as malignant preoperatively [2,4,5]. As a result of this diagnostic difficulty, OCC is frequently confused with other lesions that share overlapping histopathological features, including verrucous carcinoma, odontogenic keratocyst, and papillary squamous cell carcinoma [3]. To date, no clearly defined clinical features have been established that reliably characterize OCC, and no specific biopsy strategy has been proposed that facilitates a definitive diagnosis or improves diagnostic yield.

This report presents a case of a maxillary gingival lesion with multiple fistulas leaking white, keratin-like material from the lesion's surface. A tissue sample, including the deeply infiltrated area of the tumor, was obtained during a biopsy, leading to the diagnosis of OCC. This case report discusses the significant clinical findings indicative of OCC and outlines the essential requirements for diagnosing OCC.

## Case presentation

**Figure 1:**
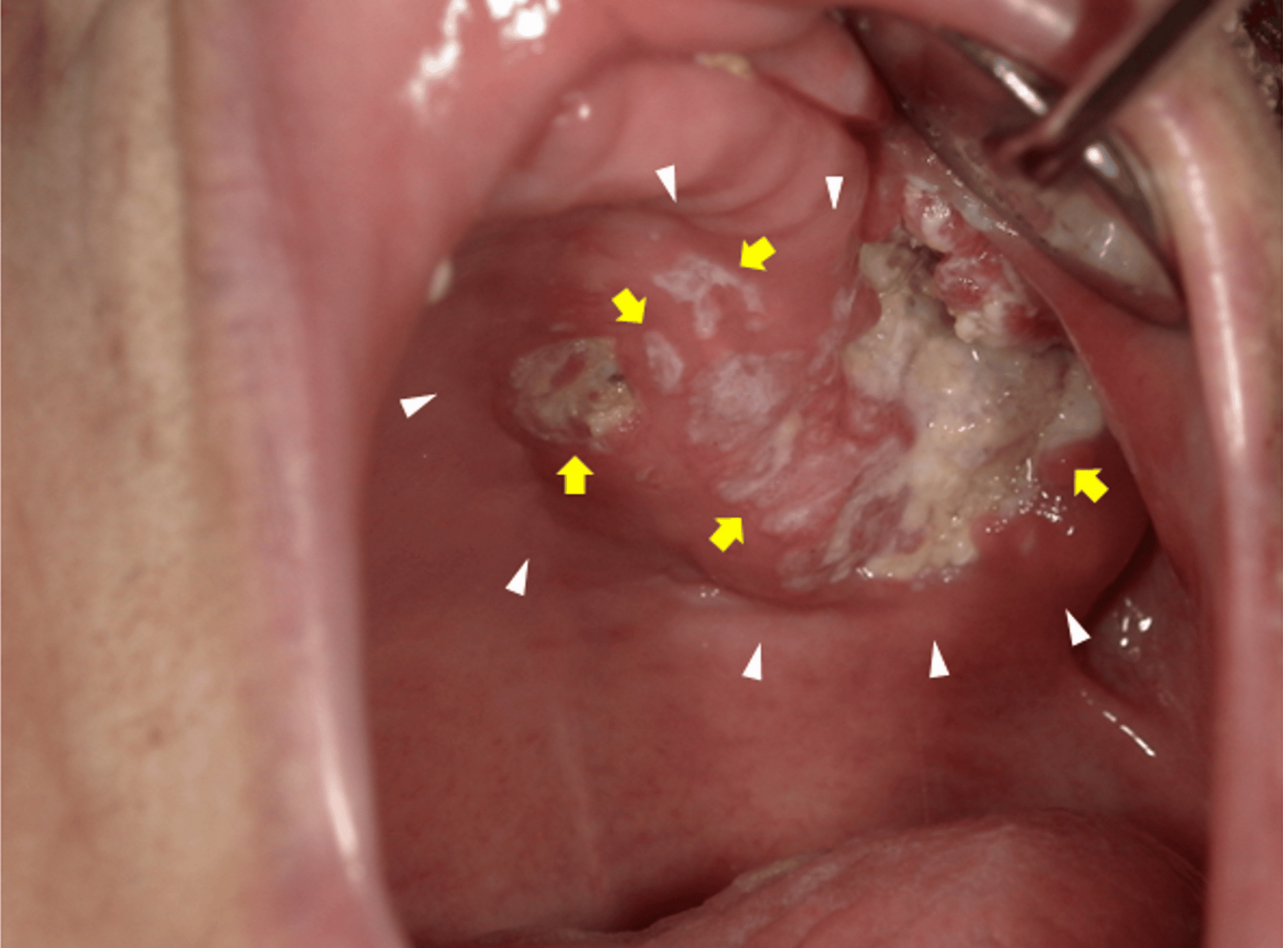
Intraoral photograph Intraoral photograph showing an exophytic lesion with multiple fistulas and white, keratin-like exudate on the surface of the left maxillary gingiva. The white arrowhead indicates the lesion, and the yellow arrows indicate the fistulas.

A 90-year-old woman presented to the department of Dentistry and Oral Surgery of Kinki Central Hospital with chief complaints of pain in the left maxillary region, along with symptoms of intermittent pain beginning one month prior. Her medical history included cerebral infarction, type 2 diabetes mellitus, hypertension, and hyperthyroidism, with no history of smoking or alcohol consumption. Intraoral examination revealed a granular endophytic lesion measuring 18×40 mm on the left side of the maxillary gingiva, characterized by multiple fistulas discharging white, keratin-like material (Figure 1).

The initial laboratory test results are summarized in Table 1, and no other abnormalities were detected in the remaining blood examinations. An elevated SCC antigen level clinically raises suspicion for the presence of squamous cell carcinoma.

**Table 1:** Abnormal laboratory findings on initial blood examination All parameters listed in the table represent abnormal findings on blood examination.

Laboratory test	Result	Reference range	Unit
Squamous cell carcinoma antigen (SCC antigen)	3.1	≤1.5	ng/mL
Alkaline phosphatase (ALP)	354	38-113	U/L
Creatinine (Cr)	1.24	0.6-1.1	mg/dL
Blood glucose	316	70-110	mg/dL
Hemoglobin A1c (HbA1c)	11	4.6-6.2	%

**Figure 2:**
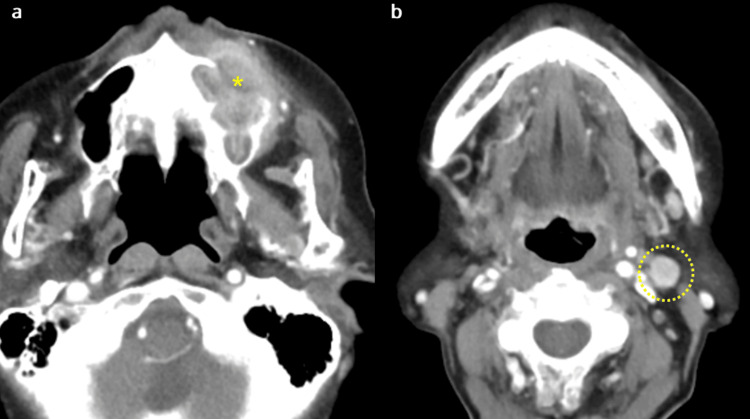
Computed tomography scan a: CT image of the left maxilla showing a lesion with a slightly indistinct margin and heterogeneous internal contrast enhancement. Marked bone resorption of the surrounding maxillary bone resulted in loss of the maxillary sinus floor (yellow asterisk). Increased density within the maxillary sinus is also observed. b: CT image showing enlargement of lymph nodes with contrast enhancement in the left submandibular lymph node.

Enhanced computed tomography revealed an area in the left maxilla with a slightly indistinct border and internal non-uniform contrast enhancement. Marked resorption of the maxillary bone surrounding the lesion resulted in the disappearance of the bone tissue on the maxillary sinus floor. Additionally, an increased density within the maxillary sinus was observed (Figure 2A). Moreover, enlargement of the left submandibular lymph node was observed, with contrast enhancement (Figure 2B).

**Figure 3:**
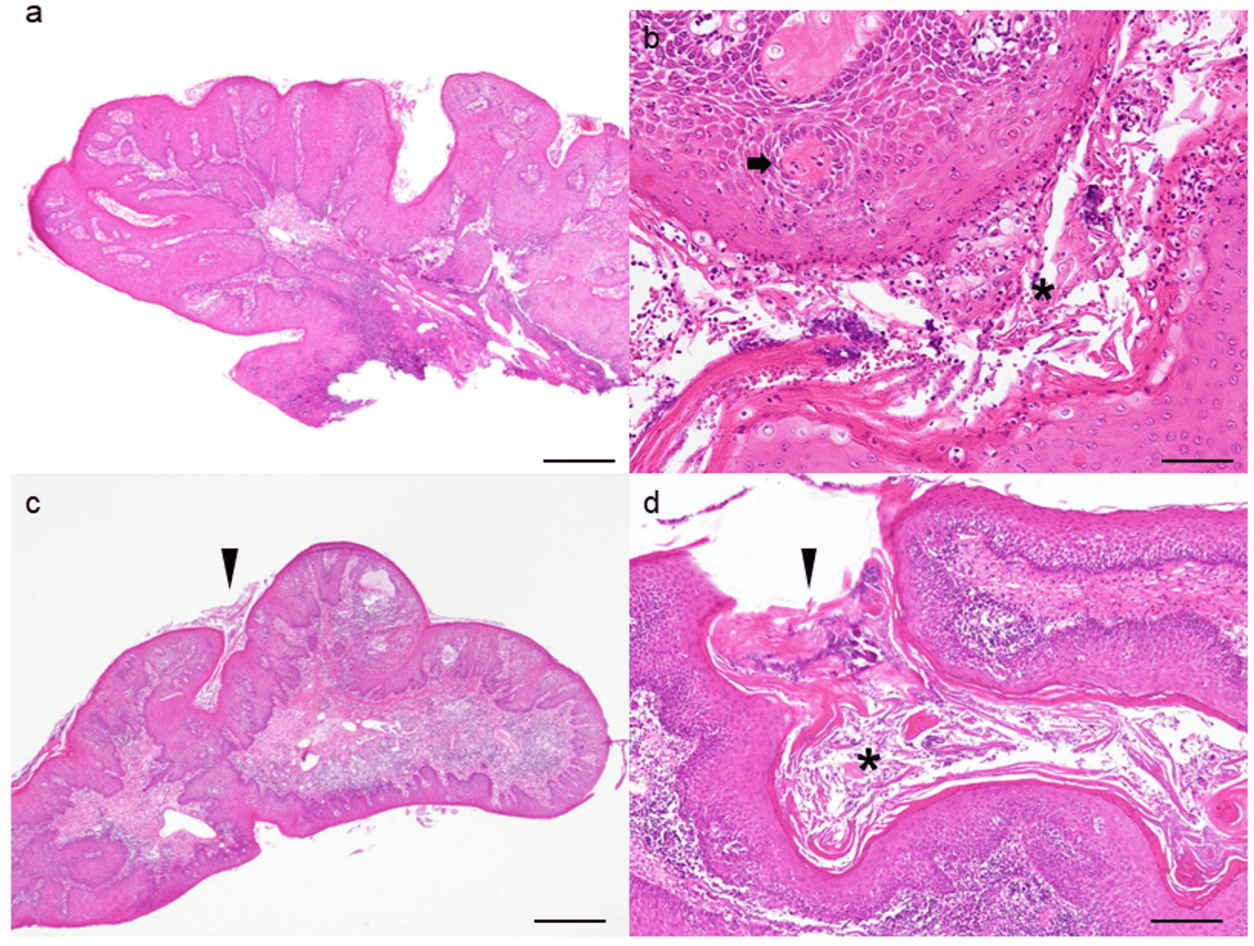
Histopathological images of the initial biopsy (a, b) and the second biopsy (c, d) a: Histopathological image of the initial biopsy showing papillomatous-like proliferation of a thickened stratified squamous epithelium with parakeratosis. Hematoxylin and eosin staining. The scale bar indicates 640 µm. b: Histopathological image of the initial biopsy showing the preservation of basal cell polarity without signs of invasion or cellular atypia. Keratin accumulation was observed between the epithelial layers, likely exfoliated from the cornified layer, accompanied by the infiltration of inflammatory cells (black asterisk). Partially formed cancer pearls were identifiable within the epithelium (black arrow). Hematoxylin and eosin staining. Scale bar indicating 100 µm. c: Histopathological image of the second biopsy showing findings similar to those in image a. The black arrowhead indicates the entrance of the cuniculatum sinus. Hematoxylin and eosin staining. The scale bar indicates 640 µm. d: Histopathological image of the second biopsy showing findings similar to those in image b. The black arrowhead indicates the entrance of the cuniculatum sinus, and the black asterisk indicates the keratin plug. Hematoxylin and eosin staining. The scale bar indicates 100 µm.

Given the clinical findings, malignancy was strongly suspected. However, cytological examination of the intraoral lesion via scraping revealed squamous epithelial cells without atypia. Furthermore, fine-needle aspiration cytology of enlarged lymph nodes in the left submandibular region did not indicate metastasis. Despite two biopsies that sampled tissue from the superficial portion of the tumor, histopathological examination revealed connective tissue fragments covered by stratified squamous epithelium with minimal cellular atypia. There was no indication of invasion or significant cellular atypicality; therefore, a diagnosis of SCC could not be confirmed (Figure 3).

Subsequently, a third biopsy was performed to obtain tissue samples from the bone resorption area, which represented the deepest part of the tumor. After a thorough preoperative evaluation of the bone morphology using computed tomography, the incision was made deeply until the scalpel blade reached the bone surface, enabling resection of tissue from the deepest portion. A single paraffin block was prepared from the biopsy specimen, and the tissue was sectioned in an orientation that allowed evaluation of the full thickness from the superficial mucosal surface to the deep portion. The biopsy specimens were fixed in 10% neutral buffered formalin and embedded in paraffin. Serial sections were prepared according to standard histopathological protocols, and multiple levels were examined. No ancillary immunohistochemical staining was performed.

**Figure 4:**
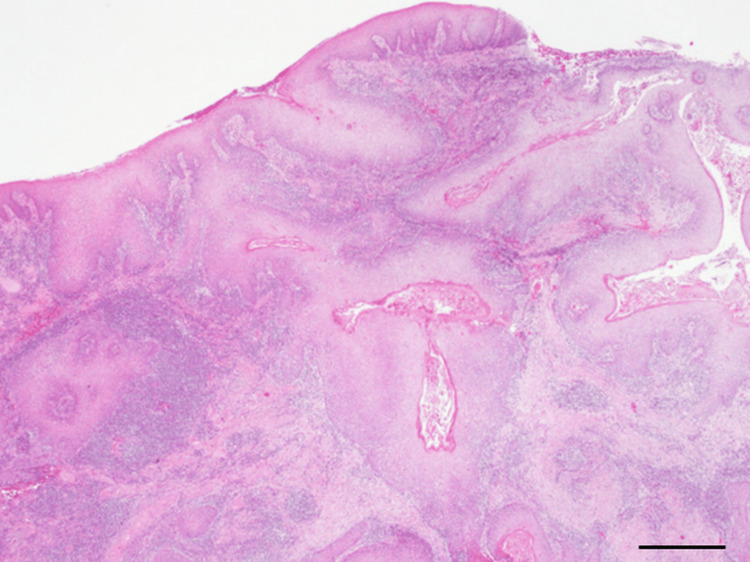
Pathological image of the third biopsy Pathological image reveals the mucosal epithelium exhibiting parakeratosis and irregular thickening, extending deeply to form branching sinuses filled with keratin. Minimal cell aberration was observed, and the polarity of the basal cell layer was nearly preserved. Hematoxylin and eosin staining. The scale bar indicates 640 µm.

In the obtained tissue specimens, the stratified squamous epithelium extended to the deepest part of the bone. The observed epithelium, exhibiting parakeratosis and partial irregular thickening, extending deeply, and forming a branching sinus filled with keratin, is known as the "cuniculatum architecture". In addition, there was minimal cellular atypia, and the polarity of the basal cell layer was nearly preserved (Figure 4).

**Figure 5:**
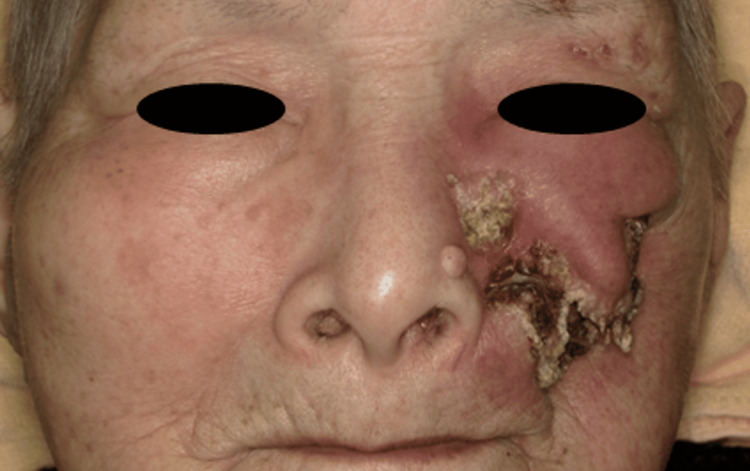
Facial photograph Facial photograph showing the tumor penetrating the cheek skin and undergoing self-destruction.

A definitive diagnosis of carcinoma cuniculatum was established based on clinical and pathological considerations. We recommended further examinations and definitive treatment; however, the patient declined these interventions, and best supportive care was initiated. At the initial presentation, the presence of the disease itself had a minimal impact on the patient's quality of life. During subsequent follow-up, the tumor progressively enlarged. Twenty-four months after the initial visit, the patient revisited the outpatient clinic, at which time the tumor had significantly enlarged, penetrating the cheek skin and undergoing self-destruction (Figure 5).

As a consequence of this tumor progression, food leakage from the oral cavity to the extraoral region developed, accompanied by the onset of pain, a pronounced malodor, and marked facial disfigurement, resulting in a substantial deterioration in the patient's quality of life, including functional, social, and cosmetic aspects.

The clinical course can be summarized as follows: initial presentation, two inconclusive superficial biopsies, a third deep biopsy guided by CT findings, followed by disease progression under best supportive care.

## Discussion

Based on the present case, we propose two important considerations regarding the diagnostic approach to OCC. First, in cases clinically suspected of OCC, a biopsy to obtain tissue samples from deeper regions of the tumor should be performed [5,6]. Second, the presence of a fistula leaking keratin-like material is an important observation when considering a diagnosis of OCC.

OCC exhibits a characteristic histopathological structure resembling rabbit burrows, with well-differentiated stratified squamous epithelium proliferating and deeply infiltrating, forming a complex pattern of keratin-filled crypts [3,7-9]. However, achieving a definitive diagnosis of OCC before surgery can be challenging, and various initial biopsy-based diagnoses fail to confirm OCC [3,8]. Two factors contributing to this diagnostic challenge have been identified [9].

The first challenge in diagnosing OCC is the limited presence of histopathological malignant findings [10]. Treating OCC as a malignant disease is difficult because the squamous epithelial cells exhibit minimal cell atypia, forming a highly differentiated and thickened stratified squamous epithelium that proliferates while maintaining basal cell polarity [11]. The second challenge stems from the inappropriate selection of the tissue sampling site during the biopsy procedure [5-8,12]. OCC is highly locally invasive and infiltrating, expanding through the formation of a tunnel within normal tissue, resulting in a structure known as the cuniculatum [8,9]. If biopsy samples are limited to the surface-level tumor tissue, capturing the characteristic cuniculatum structure of OCC becomes challenging. Moreover, the presence of squamous epithelium with pronounced keratinization and hypertrophy can resemble benign tumors such as squamous papilloma or low-grade squamous tumors, including verrucous carcinoma, papillary squamous carcinoma, or odontogenic keratocysts [5-7,12-16].

We believe that the key to diagnosing OCC lies in capturing the proliferation and invasion of the highly differentiated stratified squamous epithelium within deep tissue, highlighting the importance of biopsy procedures for obtaining tissue samples from invasive sites.

However, although sampling tissue from the invasive front is essential for an accurate diagnosis, accurately identifying this front on conventional imaging modalities remains difficult. Likewise, during biopsy procedures, obtaining sufficiently deep tissue that truly represents the invasive front is often challenging when the biopsy is performed from the superficial mucosal surface.

OCC is known to show a strong tendency for bone invasion [3]. Accordingly, tumor tissue located adjacent to radiographically detected bone resorption can reasonably be considered to represent the invasive front. Recent advances in cone-beam computed tomography (CBCT) enable precise three-dimensional evaluation of maxillofacial bone morphology, facilitating accurate identification of osseous structures and areas of bone alteration [17,18].

In this context, obtaining biopsy specimens from tumor tissue adjacent to bone, using the bone surface as an anatomical landmark, may represent a feasible and practical approach. Thus, biopsy sampling from tumor tissue adjacent to bone, guided by the bone surface as an anatomical landmark, may be a practical strategy. Advances in three-dimensional maxillofacial imaging allow accurate anatomical guidance, indicating that image-guided or navigation-assisted biopsy may improve sampling accuracy at invasive sites [19].

In this case, the initial two biopsies were performed to obtain a sample with a limited surface area and failed to attain a conclusive diagnosis. Consequently, a third biopsy was performed after a thorough evaluation of bone morphology based on CT imaging data, ensuring that the scalpel had reached the deepest area of bone involvement.

The crucial element in conducting a proper biopsy is the clinician's ability to suspect OCC. OCC presents as a primary lesion characterized by endophytic growth and mild exophytic proliferation accompanied by surrounding induration. Moreover, the superficial layer frequently presents as a cobblestone-like pink-to-red or white lesion [3,7]. Although this lesion, occasionally associated with bone invasion, is commonly perceived as a malignant condition by clinicians, the failure to suspect OCC is assumed to lead to improper tissue collection during biopsy procedures.

To suspect OCC, it is desirable to identify the characteristic findings associated with OCC; however, there have been no definitive reports outlining these findings. OCC forms a continuous crypt extending from the surface to the interior, abundant in keratin, and occasionally contains pyogenic products resulting from microinfections [3,20]. Various studies have documented the presence of multiple orifices on the surface discharge of foul-smelling white to yellowish substances composed primarily of keratin and pus [4,5,7,9-11,16,21]. We consider the existence of this keratin-leaking fistula to be a significant finding in suspected OCC. In this case, multiple fistulas formed on the lesion's surface, releasing a viscous, white, keratin-like substance. This observation was notable when gentle pressure was applied to the tumor.

While identifying fistulas with keratin-like material leakage on the lesion surface has been observed in previous OCC reports, it is not considered a universal finding at this stage. Additionally, this manifestation is not exclusive to OCC, as it can be seen in other conditions characterized by keratin shedding from the tumor epithelium, such as verrucous carcinoma and odontogenic keratocysts.

Several limitations of this report should be acknowledged. First, whole-body imaging was not performed because the patient declined further diagnostic examinations, which limited the assessment of regional or distant metastasis and precluded accurate clinical staging. In addition, follow-up imaging was not available. Second, this report describes a single case, and the findings should therefore be interpreted with caution. Although the presence of fistulous tracts with keratin-like discharge has been reported in previous cases of OCC, this feature cannot currently be regarded as a universal finding. Moreover, this manifestation is not specific to OCC, as similar keratin leakage may also be observed in other lesions characterized by keratin shedding from the tumor epithelium, including verrucous carcinoma and odontogenic keratocysts.

## Conclusions

In conclusion, this case highlights the diagnostic challenges of OCC. The present findings further support the importance of considering OCC when encountering endophytic lesions with keratinous fistulas and underscore the value of obtaining biopsy specimens from deeper invasive regions. Close collaboration between clinicians and pathologists remains essential for accurate recognition of this rare carcinoma and for guiding appropriate clinical management.

## References

[1] Flieger S, Owiński T (1977). Epithelioma cuniculatum an unusual form of mouth and jaw neoplasm (in Polish). Czas Stomatol.

[2] Huang PJ, Ting CH, Leu YS, Ho NH, Lin CF (2025). Oral carcinoma cuniculatum: a rare, clinically challenging entity-a case report and updated systematic review. Head Neck.

[3] Yadav S, Bal M, Rane S, Mittal N, Janu A, Patil A (2022). Carcinoma cuniculatum of the oral cavity: a series of 6 cases and review of literature. Head Neck Pathol.

[4] Nagai M, Pastwik B, Aguirre A, Burke M, Campbell J (2023). Carcinoma cuniculatum: a rare malignancy with unique diagnostic dilemmas. Cureus.

[5] Ajith A, Subramaniam N, Balasubramanian D, Thankappan K, Iyer S (2018). Carcinoma cuniculatum of the oral cavity: a diagnostic dilemma. J Head Neck Physicians Surg.

[6] Thavaraj S, Cobb A, Kalavrezos N, Beale T, Walker DM, Jay A (2012). Carcinoma cuniculatum arising in the tongue. Head Neck Pathol.

[7] Padilla RJ, Murrah VA (2014). Carcinoma cuniculatum of the oral mucosa: a potentially underdiagnosed entity in the absence of clinical correlation. Oral Surg Oral Med Oral Pathol Oral Radiol.

[8] Farag AF, Abou-Alnour DA, Abu-Taleb NS (2018). Oral carcinoma cuniculatum, an unacquainted variant of oral squamous cell carcinoma: a systematic review. Imaging Sci Dent.

[9] Pons Y, Kerrary S, Cox A (2012). Mandibular cuniculatum carcinoma: apropos of 3 cases and literature review. Head Neck.

[10] Sun Y, Kuyama K, Burkhardt A, Yamamoto H (2012). Clinicopathological evaluation of carcinoma cuniculatum: a variant of oral squamous cell carcinoma. J Oral Pathol Med.

[11] Ourania S, Olga B, Vasiliki K (2023). Carcinoma cuniculatum of the maxilla mimicking nonhealing extraction sockets at the right molar region - an interesting case. Eplasty.

[12] Fonseca FP, Pontes HA, Pontes FS (2013). Oral carcinoma cuniculatum: two cases illustrative of a diagnostic challenge. Oral Surg Oral Med Oral Pathol Oral Radiol.

[13] Suzuki J, Hashimoto S, Watanabe K, Takahashi K, Usubuchi H, Suzuki H (2012). Carcinoma cuniculatum mimicking leukoplakia of the mandibular gingiva. Auris Nasus Larynx.

[14] Sivapathasundharam B, Kavitha B, Padmapriya VM (2018). Carcinoma cuniculatum of the alveolar mucosa: a rare variant of squamous cell carcinoma. Head Neck Pathol.

[15] Murai C, Sakata KI, Ouchi C (2022). Mandibular carcinoma cuniculatum around the dental implant in a patient with concurrent management for pemphigus vulgaris: a case report. Oral.

[16] Janardhanan M, Rakesh S, Savithri V, Aravind T, Mohan M (2021). Carcinoma cuniculatum of mandible masquerading as odontogenic keratocyst: challenges in the histopathological diagnosis. Head Neck Pathol.

[17] Alhawasli RY, Ajaj MA, Hajeer MY, Al-Zahabi AM, Mahaini L (2022). Volumetric analysis of the jaws in skeletal class I and III patients with different facial divergence using CBCT imaging. Radiol Res Pract.

[18] Hajeer MY, Al-Homsi HK, Alfailany DT, Murad RM (2022). Evaluation of the diagnostic accuracy of CBCT-based interpretations of maxillary impacted canines compared to those of conventional radiography: an in vitro study. Int Orthod.

[19] Jaber ST, Hajeer MY, Khattab TZ, Mahaini L (2021). Evaluation of the fused deposition modeling and the digital light processing techniques in terms of dimensional accuracy of printing dental models used for the fabrication of clear aligners. Clin Exp Dent Res.

[20] Puxeddu R, Cocco D, Parodo G, Mallarini G, Medda M, Brennan PA (2008). Carcinoma cuniculatum of the larynx: a rare clinicopathological entity. J Laryngol Otol.

[21] Shapiro MC, Wong B, O'Brien MJ, Salama A (2015). Mandibular destruction secondary to invasion by carcinoma cuniculatum. J Oral Maxillofac Surg.

